# On the prediction of premature births in Hispanic labour patients using uterine contractions, heart beat signals and prediction machines

**DOI:** 10.1049/htl2.12044

**Published:** 2023-04-08

**Authors:** Ejay Nsugbe, Jose Javier Reyes‐Lagos, Dawn Adams, Oluwarotimi Williams Samuel

**Affiliations:** ^1^ Nsugbe Research Labs Swindon UK; ^2^ School of Medicine Autonomous University of Mexico State (UAEMéx) Toluca de Lerdo Mexico; ^3^ School of Computing Ulster University Newtownabbey UK; ^4^ School of Computing and Engineering Derby University of Derby Derby United Kingdom

**Keywords:** support vector machines, physiological models, signal classification, medical signal processing, learning (artificial intelligence), decision support systems, biocybernetics, medical control systems

## Abstract

Preterm birth is a global epidemic affecting millions of mothers across different ethnicities. The cause of the condition remains unknown but has recognised health‐based implications, in addition to financial and economic ones. Machine Learning methods have enabled researchers to combine datasets using uterine contraction signals with various forms of prediction machines to improve awareness of the likelihood of premature births. This work investigates the feasibility of enhancing these prediction methods using physiological signals including uterine contractions, and foetal and maternal heart rate signals, for a population of south American women in active labour. As part of this work, the use of the Linear Series Decomposition Learner (LSDL) was seen to lead to an improvement in the prediction accuracies of all models, which included supervised and unsupervised learning models. The results from the supervised learning models showed high prediction metrics upon the physiological signals being pre‐processed by the LSDL for all variations of the physiological signals. The unsupervised learning models showed good metrics for the partitioning of Preterm/Term labour patients from their uterine contraction signals but produced a comparatively lower set of results for the various kinds of heart rate signals investigated.

## Graphical abstract

Decision support model using uterine contractions and for the first time ever, using maternal and foetus heart rate signals.

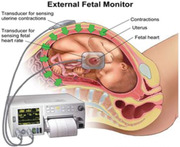

## 1 INTRODUCTION AND BACKGROUND

Preterm has long been viewed as a global scale epidemic which is one of the leading causes of death of infants, and where the select infants who survive the infancy period are left with various lifelong ailments because of their reduced timeframe in the womb during gestation [1]. The World Health Organization (WHO) guidelines characterise a preterm as birth before 37 weeks of gestation, 37–42 weeks is term, and over 42 weeks as post term [2].

The underlying causes and physiological manifestations of preterm births remain the subjects of continuous research, where known causes of premature delivery include early induction of the pregnant mother for safety reasons, rupture of the membrane, as well as spontaneous contractions [3]. To a degree, factors such as a rupture of the blood vessels, congenital deformities and overall weakness of the cervix have also been seen to cause early and preterm delivery in pregnant mothers [1]. Other subtle maternal factors which have been seen to correlate towards preterm births include short intervals between births, age, low BMI, as well as chronic stress, high alcohol consumption and smoking [1].

There also exist financial implications of a preterm birth, the magnitude of which vary depending on the severity of the preterm delivery, while statistics from England and Wales suggest costs of care of a preterm individual all the way towards adulthood are in the range of £62 000–£95 000 [1, 4, 5]. An added source of the societal cost of preterm also stems from false positive diagnoses due to unreliable means for the prediction of premature pregnancies and has been seen to result in a cost of approximately $20 000 per patient [1, 4, 5].

Current means towards detecting preterm pregnancies include an estimation of the length of the cervix, which has been seen to be linked towards the duration of pregnancy, and where ultrasound measurement is used as the auxiliary measurement tool for this method; but this approach has been seen to be inaccurate due to its qualitative nature [6]. The use of biochemical markers from emissions from biological fluids such as urine, cervical mucus and saliva have been tested as a means towards predicting preterm births to limited success, with the overarching conclusion being that even a combination of biomarkers is still insufficient for a reliable prediction of a premature birth and delivery [7, 8, 9]. More recently, methods analysing the contraction patterns and frequency of the womb have been used, and highly favoured, as this allows for a continuous means and platform towards pregnancy monitoring, which can reduce hospital visits and improve the swiftness in picking up anomalous incidents during the gestation period [4, 10]. For this approach, measurement tools such as electrohysterogram/uterine electromyography, which records bio‐electric signals, and tocogram, which records mechanical displacements from womb‐based contractions, are commonly used tools in this area, where the challenges associated with this include identifying effective decoding and signal processing algorithms to interpret contraction patterns [3, 4, 10].

**FIGURE 1:**
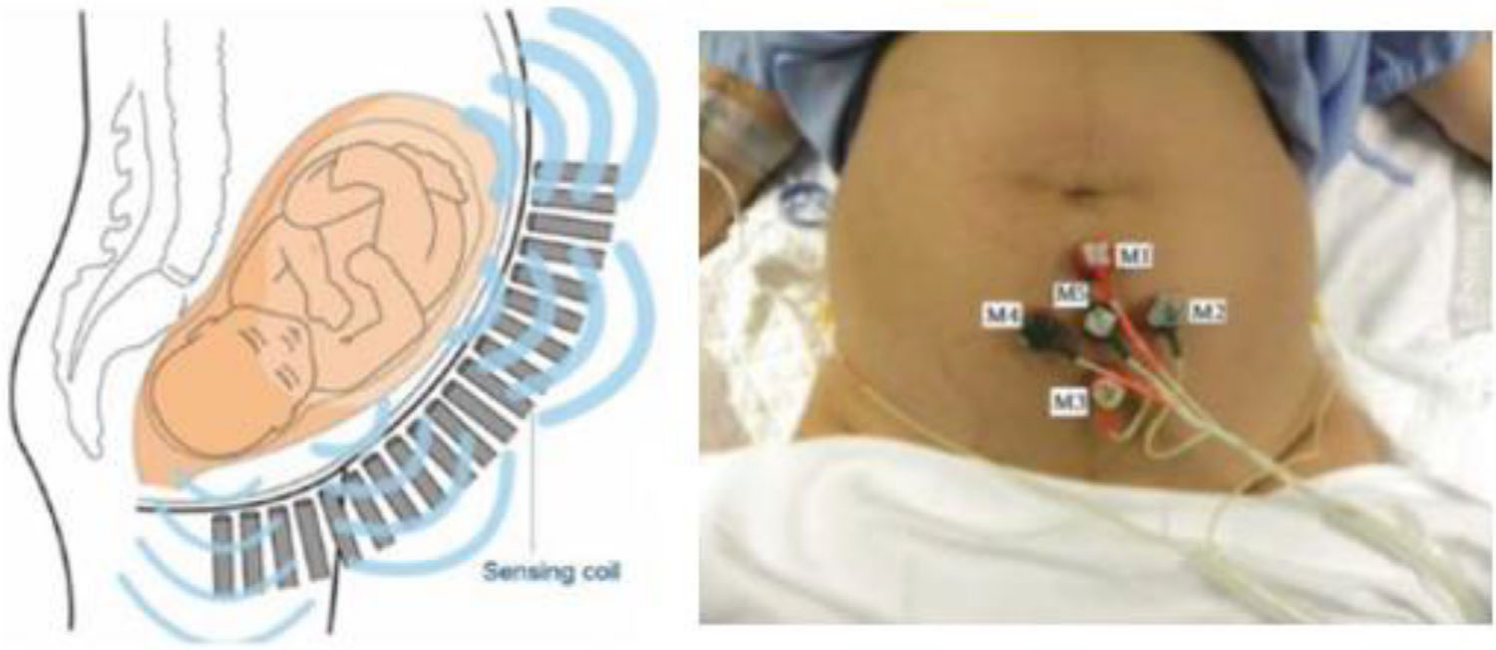
Images showing various acquisition settings and protocols from related literature [17, 18].

In terms of uterine contractions, there has been a surge of research work using machine learning models towards the development of pattern recognition algorithms that can proactively aid in the diagnosis of preterm predictions [3, 11, 12, 13, 14, 15, 16]. These works use contraction signals from patients who are in varied points of the third trimester of pregnancy, when distinct contraction signals begin to occur [3, 11, 12, 13, 14, 15, 16]. The results from these model‐based prediction exercises showed vastly impressive results for an array of signal processing, particularly non‐linear signal processing and machine learning models. However, as noted by Nsugbe et al. [3], much of the published literature in this area has focused on tuning and optimization of the pattern recognition models used for the identification and distinguishing of the various pregnancy states, with sparse considerations of how this would fit into a clinical setting with input from clinical experts, enabling applied human‐machine interaction. An image of the various signal acquisition setups used in the collection of the uterine contraction signals with various physiological instruments can be seen in Figure 1.

In work undertaken towards tackling the shortcoming in published literature, Nsugbe et al. adopted principles of hierarchical cybernetics towards the theoretical assembly of a cybernetic system which hosts a prediction machine [3, 19]. This subsequently feeds its decisions and predictions to the clinical experts in the loop, who make the final decision on the treatment and care option for the patient given the immediate evidence from expert knowledge, as well as the predictions made by the model in the loop, forming a human‐machine decision platform [3, 19]. The source of the information used by the prediction machine varied between electrohysterogram (EHG)‐tocogram and magnetomyography (MMG) data via open‐source pregnancy databases [20, 21].

Recent work by López‐Justo et al. [22] involved the collection of data from a group of Hispanic patients in active labour, where specific emphasis was placed on the analysis of both the foetal and maternal heart rates. In that study, a link was seen to exist between the beat‐to‐beat heart rate signals and the delivery of term/preterm foetuses, using a complexity theory approach [22].

This work aims to use a range of machine learning to quantify the extent to which these signal sources can be used to predict preterm deliveries for a group of Hispanic women in active labour. This work also presents a novel insight into preterm prediction exercises with data collected from a large group of Hispanic women based on published research. Thus, explicitly speaking, the contributions of this research paper are as follows:

- Preterm prediction of Hispanic women in active labour using the Raw acquired EHG, Foetus Beat‐to‐Beat Signal, and Maternal Beat‐to‐Beat Signal;
- Preterm prediction of Hispanic women in active labour using EHG, Foetus Beat‐to‐Beat Signal, and Maternal Beat‐to‐Beat Signal alongside the Linear Series Decomposition Learner;
- Comparison of the Machine Learning model prediction performance across the following supervised learning machine learning algorithms: decision tree, linear discriminant analysis, logistic regression, support vector machines (i.e. linear SVM), quadratic SVM, cubic SVM, fine Gaussian SVM, and k‐nearest neighbour;
- Pilot exploration and contrast on the use of self‐learning unsupervised algorithms observing the self‐sorting of pregnancy states purely from physiological data.

## 2 MATERIALS AND METHODS

### 2.1 Data collection

The data used as part of this study is from the published work of López‐Justo et al. [22], where physiological readings and recordings were collected from patients at the “Mónica Pretelini Sáenz” Maternal‐Perinatal Hospital, Toluca, State of Mexico, Mexico, where the study received ethical consent by the local ethical committee, and informed consent was obtained from all patients prior to the collection of the data. Those patients clinically identified as being in preterm labour were between 32–36 weeks' gestation, with term labour occurring between 38–40 weeks' gestation [22]. Active labour was clinically identified as the onset of four distinct contractions within a period of 10 minutes, and cervical effacement of 50% with an accompanying 4 cm of cervical dilation [22]. Women who had twin pregnancies, gestational diabetes, hypertensive disorders, epidural anaesthesia, or chronic degenerative diseases were excluded from recruitment [22].

Data was collected from term patients and preterm patients where the heartbeat signals from both the foetuses and mother were recorded using the Monica AN24 system, designed by Monica healthcare [23]. This is a system with the ability to record foetal cardiac time intervals to a high precision, where abdominal cardiogram signals have been seen to offer more reliable recording of the foetal heart rate when compared with cardiotocography [23]. The EHG data was recorded using electrodes in a bipolar configuration, where alcohol was used to cleanse and prepare skin beforehand to minimize potential impedance. The sampling frequency for all acquisitions was seen to be 900 Hz.

The characteristics of both patient cohorts and foetuses can be seen in Table 1.

**TABLE 1:** Characteristics of both patient cohorts and their delivered foetuses

Characteristic	Term	Preterm
Maternal age (years)	21 ± 4	21 ± 4
Weeks of gestation	39 ± 1	34 ± 2
Maternal BMI (kg/cm^3^)	24.3 ± 1.3	25 ± 2.8
Cervical dilation (cm)	5.9 ± 1.6	5.0 ± 1.7
Cervical effacement (%)	71 ± 12	62 ± 13
New‐born birth weight (kg)	2.9 ± 0.4	2.4 ± 0.6
APGAR score 1 min (>7)	96%	80%
APGAR score 5 min (>7)	96%	70%
Head circumference (cm)	33.7 ± 1.73	32.0 ± 2.29
Foetal size (cm)	49.5 ± 2.1	45.1 ± 6.0
Gender percentage	52%	50%
R‐R means (ms)	431.2 ± 31.0	413.2 ± 26.9

### 2.2 Signals

#### 2.2.1 EHG

The resulting contraction signals from the uterine wall during pregnancy—especially during the third trimester—have been seen to be linked towards the overall state of the pregnancy, where the dynamic behaviour of the waveform is dependent on the phase and cycle of the gestation, in addition to several other physiological factors [24, 25]. On a cellular scale, these bioelectrical contraction signals can be described by the Hodgkin‐Huxley electrophysiological model, where cellular depolarization takes place above a set threshold and yields an accompanying electrophysiological burst event which is linked to pregnancy state, but also influenced by a number of factors [24, 25].

#### 2.2.2 Heartbeat signals

**FIGURE 2:**
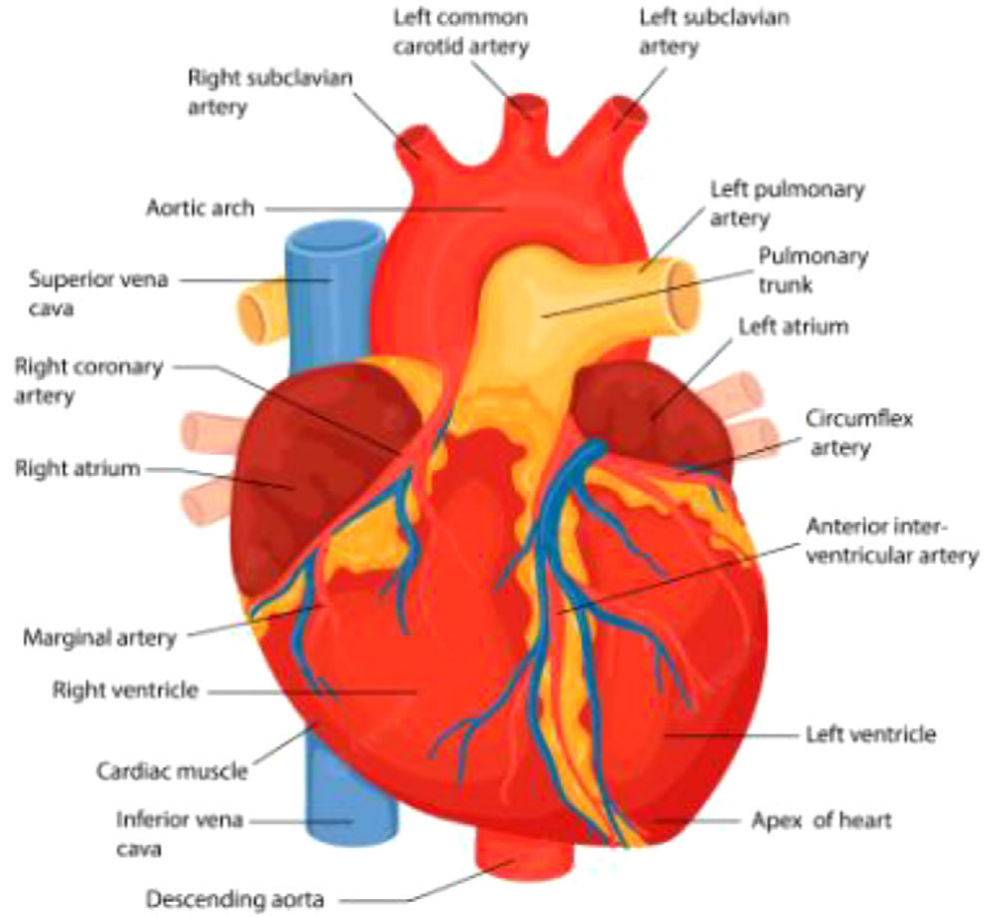
Annotated image of the human heart [27].

The measure of the heart rate is referred to as the heart rate variability, which quantifies the amount of time elapsed between successive heartbeats, and can be a window towards a physiologically driven means of diagnosing not only heart‐based problems but also psychological issues such as mental illness and psychiatric disorders [26]. The normal baseline breathing of a human being is termed as sinus rhythm, and its name is reflected in the fact that the human heartbeat is influenced by the respiratory system and is in tandem with the reflex of the heart and circulatory system [26]. An image of the human heart can be seen in Figure 2.

The human heart has an intrinsic baseline which varies depending on the activity being carried out at the time, where a relaxed state is reflected with a slower heartbeat, whereas a faster heartbeat frequently reflects a state of stress and/or danger [26]. There also exist times where the heart rate varies due to the needs of the human body, that is, postprandially, during periods of exercise, during pregnancy, and as a natural part of the aging process [26]. The brain controls the heart rate in relation to those signals it receives as part of the autonomic nervous system. This feedback loop is controlled with the aid of nerves which serve as information carriers [26].

#### 2.2.3 Foetal heart rate (FHR)

The heartbeat of a foetus can be heard around six weeks into the gestation during a sonomyography scan. The heartbeat can be anticipated to be around 110 beats per minute, increasing to around 150–170 beats per minute in and around 10 weeks of gestation [28].

The foetal heart and circulatory system develop throughout the three trimesters as part of the maturation process. During the first trimester, blood vessels form inside the embryo which eventually grow and develop into the heart and circulatory system [28]. This initially resembles a tube, which develops to form the heart and valves, with circulatory development continuing until the end of the first trimester, when bone marrow and red blood cell formation commences [28].

During the second trimester, at approximately 17 weeks gestation, the heartbeat of the foetus shifts from a spontaneous to a regulated rhythm due to the ongoing developmental activity the brain [28]. As part of this rhythm, newly formed capillaries deliver different types of blood oxygenated to the various parts of the foetus's body, while deoxygenated blood is returned to the placenta for oxygenation via the umbilical arteries [28]. Congenital heart defects may be identified and picked up during this trimester using ultrasound instrumentation. During the third trimester, the heart has sufficiently matured to self‐sustain with increased ability to function successfully outside the womb, in preparation for birth [28].

**FIGURE 3:**
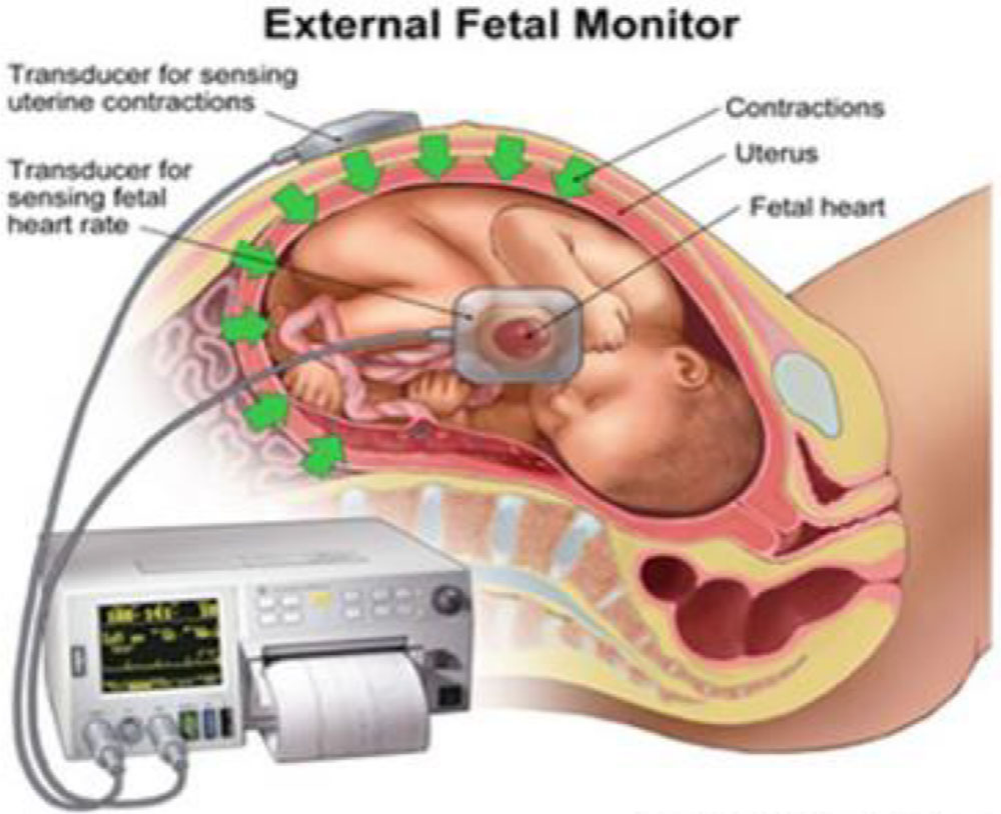
An illustration of simultaneous monitoring of both foetus heartbeat and uterine contraction [29].

An illustration of cardiotocography simultaneously recording the foetus heart and uterine contractions can be seen in Figure 3.

The heart of the foetus has been acknowledged to work differently within the uterus relative to when the baby has been born. As the foetus does not breathe oxygen directly in the womb, the lungs are closed prior to birth. The circulatory system relies on the oxygenated blood carried in the vein of the umbilical cord for the steady supply of nutrients [28]. The veins and arteries of the foetus transport nutrients and waste materials back to the mother for excretion and removal along the arteries of the umbilical cord [28].

#### 2.2.4 Maternal heart rate (MHR)

As part of the female physiological system adaptations to carrying a foetus, the cardiovascular system goes through a degree of adaptation whilst maintaining a functional baseline based on haemodynamic demands [30, 31, 32]. Further, the resting heart rate of a pregnant patient has been identified as around 15 beats per minute greater than their non‐pregnant counterparts, with an associated increase in stroke volume due to the increased maternal blood volume [30, 31, 32].

A reduction in peripheral vascular resistance, as well as the emergence of low resistance circuits to the placenta, leads to a decrease in the resting arterial blood pressure up until the end of the second trimester of pregnancy [30, 31, 32]. Around 10–20 weeks into the gestation process, blood volume begins to increase in constituents, which has been approximated to about 1500 ml [30, 31, 32].

The resting cardiac output—which is a product of stroke volume and heart rate—increases at around the fifth week of gestation and continues to steadily rise, where it peaks in the third trimester at around 30–50 % above that of a non‐pregnant patient [30, 31, 32]. The resting heartbeat increases further during the latter part of the third trimester as labour approaches, with a distinct sense of heightening occurring during the labour process, due to a combination of factors including intense uterine muscle contractions, bodily exertion, physical pain and resultant activation of the sympathetic nervous system [30, 31, 32].

A good level of fitness during pregnancy may help to ease the transition to a higher cardiac baseline and thus it is encouraged for physical activity to be maintained antenatally as reduced fitness could lead to a higher level of cardiovascular strain during the delivery process, and as a result could present an avenue for cardiac‐based disease [30, 31, 32]. The maternal heart rate can be viewed as a window towards assessing the level of strain encountered during the labour process, where the heart rate reflects an increment in the demand for oxygen due to rapid contractions of the uterine muscles, as well as the demands from the striated muscles, and isometric contractions during pushing, which is why Valsalva manoeuvres have fallen out of favour [30, 31, 32].

### 2.3 Dataset and pre‐processing

The uterine contraction signals were obtained from the MonicaSDK software, which is the supporting post‐processing software associated with the physiological instrumentation. From this, data from a single optimal channel was exported to produce an envelope—and therein a down‐sampled version of the uterine contraction signal—with a 2‐second epoch averaging scheme. The final exported files from the software varied in length due to the duration of the data collected from the various women, thus the files were down‐selected to ensure that 4 seconds worth of uterine contraction data was available for the files which were used for the analysis. Using this selection criteria, a total of 47 patients’ data was used for the final signal processing exercise, with 27 of them being term and 20 being preterm. The SMOTE synthetic sample generation algorithm was used for class balancing purposes [33].

For the heart rate variability (Maternal and Foetus), the filtered beat‐to‐beat signal was exported from the MonicsSDK software where, like the uterine contraction, an optimal channel was exported, but this time with the readings in milliseconds. From the exported signals, select patient data files which had a minimum of 3 ms in the case of the MHR and 4 ms for the FHR were used, therein leading to 46 patients’ data used for the maternal heart beat (22 preterm and 26 term), and 45 patients (17 preterm and 28 term) for the foetus heartbeat.

For both the uterine contraction signals and the various heartbeat signals, a windowing scheme of 10 disjointed windows was used, which divided each candidate signal into 10 equal windowed slices.

### 2.4 Signal decomposition method

Signal decomposition methods are signal processing methods which contribute towards a systematic separation of parts and components of a signal in order to minimise redundancy and boost overall signal quality [34]. Different kinds and modes of signal decomposition methods exist, while in this work we applied the Linear Series Decomposition Learner (LSDL) which stems from the area of metaheuristics; a subset of Artificial Intelligence. Using a set of heuristics and a linear basis function, it is capable of iteratively decomposing a signal in order to obtain an optimal region, which maximises the information quality embedded within a signal while minimising redundancy in the process [35, 36, 37, 38].

The original inception case study for the LSDL was honed towards source separation for a powder mixture using structural borne high frequency acoustic emission signals, in order to infer and estimate the particle size distribution of a sample heterogenous mixture, where the decomposition prowess and prediction performance was seen to surpass that of the wavelet decomposition method when dealing with non‐linear and stochastic signals [36, 39, 40, 41]. Recent published work has seen application in the analysis and decomposition of physiological signals from various aspects of clinical medicine including rehabilitation, preterm pregnancy, surgical anaesthesia and psychiatric medicine, all with the inclusion of the LSDL as a pre‐processing step and mechanism prior to modelling and prediction [15, 42, 43].

The embedded cost function employed as part of the evaluation of the decomposed series to assess the information quality and discriminatory power of a decomposed section of a signal was the normalised variant of the Euclidean distance which—given two sets of samples from different classes—estimates the separation between the two samples in Euclidean space [44]. The mathematical formulation for the normalised variant of the Euclidean distance is as shown in Equations (1)–(3):

(1) $$ED\;\left(p,q\right)=\sqrt{{\left(p_{1}-q_{1}\right)}^{2}+{\left(p_{2}-q_{2}\right)}^{2}\;}\;,$$

(2) $$\sigma \;=\sqrt{\frac{\sum_{w=1}^{N_{w}}{\left(r_{w}-\mu \right)}^{2}}{N_{w}}},$$

(3) $$J\;=\left(p,q\right)\;=\;\frac{ED\left(p,q\right)}{\sigma_{m}},$$

where ED is the Euclidean distance given coordinates *p* and *q*, *w* is the *w*th feature in a feature vector $N_{w}$, while$\;r_{w}$ is a specific feature within a feature vector, μ is the mean of the features, and $\sigma_{m}$ is the mean of the standard deviations of the various features being considered.

Further insight and description of the LSDL can be seen in Nsugbe and Sanusi [15] and Nsugbe et al. [42].

### 2.5 LSDL Optimal threshold seeking results for EHG, MHR and FHR

For the search of the optimal threshold regions for the various signals listed in Tables 3, 4, 5, four iterations per threshold were applied for the decomposition of the signals in both the upper and lower threshold regions, which resulted in a total of 24 iterations across the three kinds of signals. The optimal threshold regions are highlighted in bold in Tables 2, 3, 4, where the optimal region for the EHG signal is seen to be within the second iteration of the lower threshold, therein implying that the medium scale amplitude events from the EHG signals carried the rich information which maximised discriminatory capabilities for the signal. The optimal region for the maternal heart rate is seen to be in the upper threshold region with the medium scale amplitudes within that region. Finally, the foetal heart rate signal results reflected that the optimal threshold region was seen to be in the upper region within the signal, insinuating that a broader dynamic range of signals are required towards obtaining a high discriminatory prowess, potentially due to the relatively low amplitude associated with this signal, as well as a generally fine scaled nature.

**TABLE 2:** Optimal threshold region for EHG

	Iteration 1	Iteration 2	Iteration 3	Iteration 4
Upper threshold	2.1231	2.1326	2.1355	2.1368
Lower threshold	2.2506	**2.3212**	2.3194	2.1926

**TABLE 3:** Optimal threshold region for MHR

	Iteration 1	Iteration 2	Iteration 3	Iteration 4
Upper threshold	2.0087	**2.7624**	n/a	n/a
Lower threshold	n/a	n/a	n/a	n/a

**TABLE 4:** Optimal threshold region for FHR

	Iteration 1	Iteration 2	Iteration 3	Iteration 4
Upper threshold	**2.1467**	2.1402	2.0947	2.1327
Lower threshold	n/a	n/a	n/a	n/a

### 2.6 Feature extraction

The list of features extracted from all the physiological signals represents a concatenation of a diverse range of features which have been used in prior study for the modelling and identification of highly variable, non‐linear and stochastic physiological signals [45]. The ensemble comprises features from statistics, frequency, as well as non‐linear and chaos‐based features which allow for a robust and effective characterisation of a signal, despite the degree of variability and underlying non‐linearity in the signal [46, 47].

The list of features is as follows: mean absolute value (MAV), waveform length (WL), zero‐crossing (ZC), slope sign change (SSC), root mean square (RMS), fourth order autoregression (AR), sample entropy (SampEN), cepstrum (Ceps), maximum fractal length (MFL), median frequency (MedFrq), peak frequency (PeakFrq), number of peaks (NP), simple squared integral (SSI), and variance (VAR). For all features which require a threshold, 1 μv was utilised, as adopted from prior studies; while in the case of entropy, 0 and 0.2 values were chosen for m and r, respectively [46, 47].

### 2.7 Machine learning models

As part of this study, supervised and unsupervised learning methods were applied as part of the pattern recognition and classification exercises. The supervised learning methods and models work with an iterative learning scheme upon being supplied with class labels; while on the other hand, the unsupervised learning is capable of automated partitioning of data clusters with respect to a performance index, and represents a greater degree of intelligence and learning, which runs off less human intervention in contrast to the supervised variant [48].

#### 2.7.1 Supervised learning

- Decision Tree (DT): is a class of grey‐box models which are underpinned by a Boolean‐like logic towards the partitioning of data into various classes using a tree and hierarchical flow fashion [49]. Their model configuration implies that they are interpretable and therein carry a level of transparency associated with their decision making process [49].
- These classification models refer to grey‐box models whose classification method is based around the use of a Boolean logic‐like approach towards the sorting of data into different classes in a tree‐like hierarchical fashion [49]. The white‐box characteristic of the DT implies that it carries interpretability, thus its decision‐making process is transparent to a degree.
- Linear Discriminant Analysis (LDA): is a statistically driven machine learning method which utilises a lower dimensional projection of the data towards the placement of class boundaries, and makes the assumption that the data is normally distributed while being Gaussian. This version of the method utilises a linear boundary towards the separation of data classes and is recognised for being computationally efficient due to its model architecture [46].
- Logistic Regression (LR): is a parametric classification model which is based upon a statistically driven framework, where the output of the classification process ranges from 0–1, with data classes determined as a function of a given threshold [50]. Due to its non‐linear and sigmoidal function, this classification model is more robust towards dealing with outliers when compared with its linear regression counterpart [50].
- Support Vector Machine (SVM): this classification model is based around the higher dimensional projection of data into a feature space where class boundaries are instilled in an iterative fashion, and subsequently followed by a downscale and downward projection of the data while preserving the structure of the class boundaries implemented from the higher dimension in a feat which is regarded as a ‘kernel trick’ [51]. Due to the iterative nature and high dimensional projection required as part of the functionality of the model, the SVM is typically viewed as a computationally intense model [51]. Four different variants of the SVM model were employed as part of this study to provide different kinds of class boundaries to the data in question, including; linear SVM‐LSVM, quadratic SVM‐QSVM, cubic SVM‐CSVM, and fine Gaussian SVM‐FGSVM.
- K‐Nearest Neighbour: represents a type of non‐parametric classification model which hinges upon a majority vote with respect to the nearest neighbours, before classes are assigned to samples [52]. In this work, K was selected to be 1, which means the process of class assignment involves a computationally efficient class assignment process, where the Euclidean distance was utilised as the chosen distance metric.

All classification models were designed using the MATLAB Classification Learner application, which—given model options—automatically tunes the hyperparameters for the optimal values as part of the training process. The models were validated at the end using the K‐fold cross validation approach, with K chosen as 10 to obtain the final performance metric for the classification model.

#### 2.7.2 Unsupervised learning

- K‐Means: involves the iterative sorting of data into various classes based on the Euclidean distance metric. The approach is based on the expectation‐maximization (EM) algorithm where the E step includes the cluster assignment process using an objective function, while the M step is the model update phase with respect to the cluster centroid [53].
- Gaussian Mixture Models (GMM): is an unsupervised learning method which is based off probabilistic reasoning as a means towards cluster and data partitioning [54]. The model builds from the K‐Means approach towards clustering and utilises a mixture of Gaussians with distinct mixture proportions, and is characterised by a distinct mean and covariance [54]. The learning process of the GMM also uses the EM in an iterative fashion alongside the maximum likelihood estimation method. The options implemented as part of the GMM in this paper involved the use of a full covariance option with a regularisation value of 0.1.

For both models, the number of clusters was selected to be two, on the basis of the knowledge that the exercise involved a binary prediction between term and preterm pregnancies.

## 3 RESULTS AND DISCUSSION

### 3.1 Supervised learning

Four key statistical metrics were utilised towards the characterisation of the performance of the various designed models, as adopted from previous work, as follows: accuracy (Acc), sensitivity (Sens), specificity (Spec) and area under the curve (AUC) [11].

#### 3.1.1 EHG

The results for the EHG signals can be seen in Table 5 for both the raw and LSDL decomposed signals where, as expected, the LSDL supersedes the raw data in predictions of term/preterm patients in active labour. The best performing models, highlighted in bold, are seen to be the LDA and logistic regression models, which are statistically driven models and therein show compatibility of the LSDL both with this kind of data, along with the aforementioned classification models. Contrasting this with prior work on the prediction of preterm using uterine contraction signals, the high prediction accuracy obtained here is due to the fact that the women are in active labour, where uterine contractions are more intense and regular than prior points within the third trimester, and produce distinct signals that can be used towards the high prediction accuracy of preterm pregnancies, as can be seen in Table 5 [3]. The immediate findings from these results suggest that the presented approach with the EHG signal could allow for an alarm‐based system which forewarns clinicians and midwives of potential preterm pregnancies in real‐time during active labour, with a high prediction power and model integrity.

**TABLE 5:** EHG results for raw data and LSDL decomposed signals

Model	Raw‐Acc (%)	LSDL‐Acc (%)	Raw‐Sens (%)	LSDL‐Sens (%)	Raw‐Spec (%)	LSDL‐Spec (%)	Raw‐AUC (%)	LSDL‐AUC (%)
DT	73.5	94	74	92	71	97	72	94
LDA	71.3	100	71	100	71	100	71	**100**
LR	71.9	100	71	100	73	100	72	**100**
LSVM	73.7	96	72	96	76	97	74	96
QSVM	82.8	99	83	99	83	99	83	99
CSVM	83.9	98	82	98	86	99	84	98
FGSVM	87.4	97	91	97	84	97	87	97
KNN	84.3	97	82	97	87	97	84	97

#### 3.1.2 MHR

The utilisation of the heart rate signal for the inference of the state of a pregnancy has not been studied deeply in the machine learning literature, while the physiological literature suggests that during the onset of labour, the maternal heart rate adapts from its baseline in order to accommodate the imminency of labour [22]. However, it is not known if the dynamics of the heart rate during a preterm labour are distinct enough from that of a mother who is in a term labour. This investigation and associated results present a data‐driven means towards the observation of the extent to which maternal heart rate dynamic differs during term and preterm labour.

The raw data, first of all, show some encouraging results in the preterm predictions from the maternal heart rate, with the best prediction in that case coming from the KNN model. Similar to the prior scenario with the EHG signals, the LSDL provides a notable increase in the prediction accuracy provided by a model upon its application, as can be seen in Table 6, where the LR an QSVM provided the best prediction accuracies in this case. The high result from the QSVM implies that non‐linear boundaries are favourable for this classification exercise to a degree, thus providing quantitative evidence to suggest that machine learning models can indeed distinguish between term and preterm labour pregnancies using acquired heart rate signals. Furthermore, it can be noted from Section 2 that the sample segment relevant towards the model build for the heart rate‐based prediction models is substantially smaller than that of the uterine contraction, implying that quicker predictions could be made in a shorter time span with less data using the maternal heart rate when compared with the uterine contraction.

**TABLE 6:** MHR results for raw data and LSDL decomposed signals

Model	Raw‐Acc (%)	LSDL‐Acc (%)	Raw‐Sens (%)	LSDL‐Sens (%)	Raw‐Spec (%)	LSDL‐Spec (%)	Raw‐AUC (%)	LSDL‐AUC (%)
DT	70	76	69	76	68	78	68	77
LDA	55	94	55	99	55	91	55	95
LR	55	98	56	100	55	97	55	**98**
LSVM	57	94	58	100	56	90	57	95
QSVM	68	97	70	100	68	96	69	**98**
CSVM	77	96	76	98	80	96	78	97
FGSVM	77	79	86	89	73	73	79	81
KNN	80	74	80	73	82	75	81	74

#### 3.1.3 FHR

Table 7 shows the results for the preterm prediction exercises using the foetal heart rate, where it can be seen that the results provide a marginal, yet important, improvement on the figures from the predictions using maternal heart rate. The LDA and LR provided the best prediction statistics, as highlighted with the AUC values. The utilisation of heart rate physiological measurement as an indicator for preterm appears to show positive results, which have been noted to produce positive results after pre‐processing with the LSDL. This can be further developed, exploited and used as a means towards not only the monitoring of the state of the heart during labour and birth for the mother and foetus, but also modelled further for the continuous monitoring and prediction of heart related conditions that could occur during labour and birth, therein forming a cardiovascular/cardiac decision support monitoring platform.

**TABLE 7:** FHR results for raw data and LSDL decomposed signals

Model	Raw‐Acc (%)	LSDL‐Acc (%)	Raw‐Sens (%)	LSDL‐Sens (%)	Raw‐Spec (%)	LSDL‐Spec (%)	Raw‐AUC (%)	LSDL‐AUC (%)
DT	66	87	65	88	67	88	66	88
LDA	62	100	62	100	62	100	62	**100**
LR	63	100	63	100	63	100	63	**100**
LSVM	61	99	61	100	62	99	61	99
QSVM	68	99	67	100	71	99	69	99
CSVM	69	98	67	98	72	98	69	98
FGSVM	75	85	90	97	68	79	79	88
KNN	73	86	69	83	79	90	74	86

As part of further work, research would involve the investigation of what specific heart rate conditions could be inferred from the obtained maternal and foetal heart rate physiological signals as part of a broader decision support platform that is capable of providing insight on both term/preterm as well as selected conditions and diseases of the heart.

**FIGURE 4:**
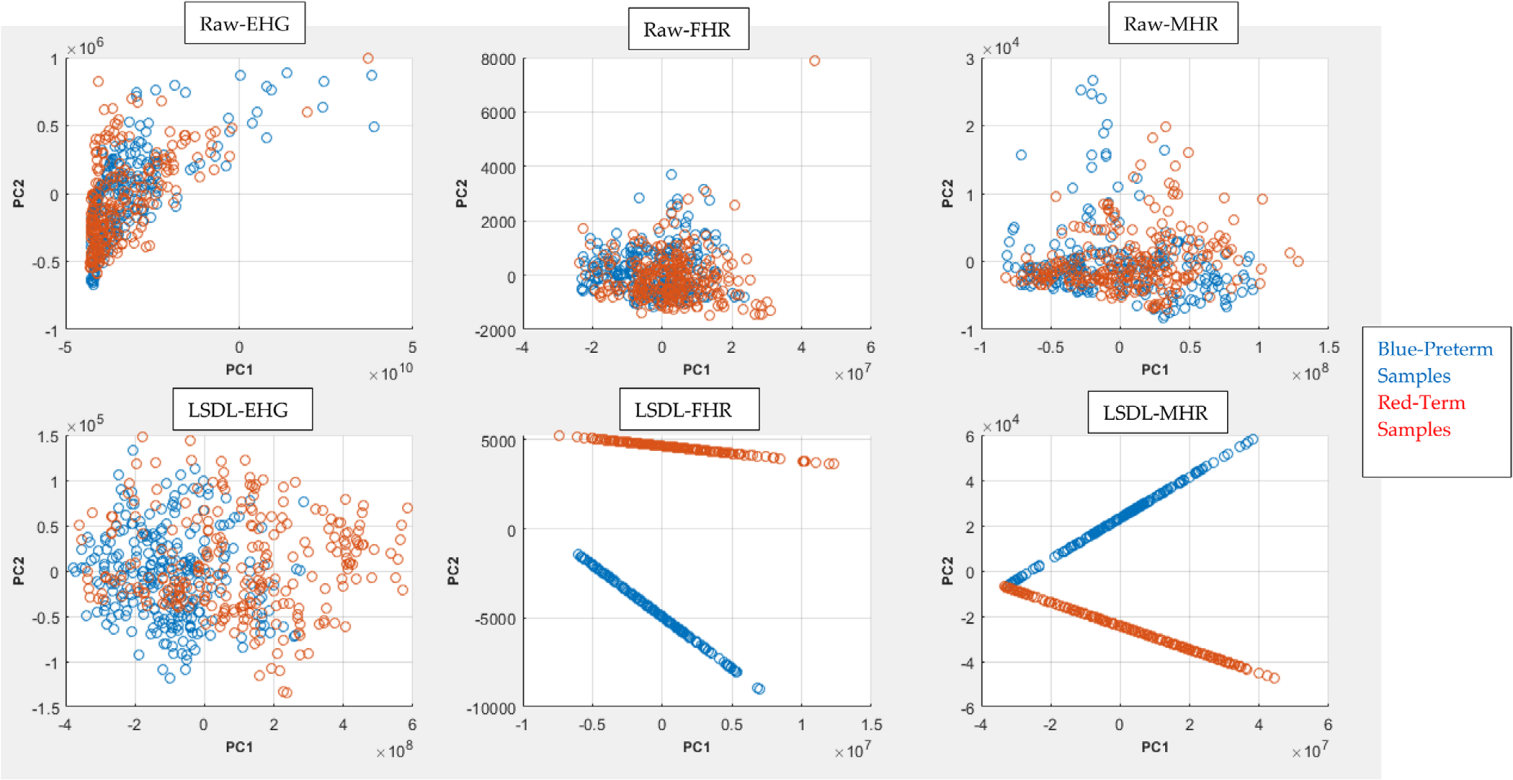
PCA visualisation plot of the various data classes.

A principal component analysis (PCA) visualisation of the data sample and class separation for all the three physiological signals considered in this study, contrasting the raw signal with the LSDL decomposed signal, can be seen in Figure 4, where the first two PCs have been plotted.

From the PCA plot, a first comparison of the Raw plot with the LSDL decomposed shows a greater cluster separability in the LSDL plot relative to the Raw, due to the signal decomposition act of the LSDL. For the LSDL FHR, a nicely decomposed and linear projection is achieved for both clusters with a notable visual separation between the two data classes, implying that the decomposed signals have low noise and a good degree of separability. In the final case of the LSDL MHR, the projections also appear to be projected nicely with minimal noise in the data post LSDL decomposition, although unlike the FHR, there appears to be a slight overlap between the two clusters.

Contrasting the three physiological signals, the heart‐based signals appear to be less noisy once decomposed by the LSDL, as can be seen and as discussed, which may present a reliable alternative towards uterine contraction‐based monitoring for preterm pregnancies. As the uterus is a complex biological system—as well as an anatomical tissue within the body—its electrophysiological signals are prone to crosstalk and biological‐based interferences, which are reflected in the acquired EHG signal. However, the dynamics of the heart are different. It is an organ which, although it is a complex system, appears to produce signals with less interferences for the prediction of preterm pregnancies. This could potentially be due to the fact that all patients were in active labour and thus the physiology was actively adapting to the labour process. Further work should involve the acquisition of the heartbeat signals earlier in the third trimester, allowing for the conducting of prediction exercises in order to further evaluate and assess the feasibility of using heartbeat signals as a reliable means towards the prediction of a preterm pregnancy.

### 3.2 Unsupervised learning

Although the results from the prior section showed largely positive results—particularly after pre‐processing with the LSDL—it is worth noting that these results were obtained using the supervised learning models which rely on the prerequisite of labelled data from expert knowledge. In a clinical setting, this accounts for further expenditure of resources; and from the viewpoint of the intelligence complexity hierarchy, it represents a form of “weak intelligence” due to the dependency of external input labels. The use of unsupervised learning represents a superior form of artificial intelligence which allows for self‐sorting and partitioning with reduced human supervision and input, albeit with the challenging task of efficient partitioning of data with complex geometries and dispersions. Tables 8, 9, 10 show the results of the use of two effective unsupervised learning methods, that is, GMM and K‐Means, towards the self‐sorting of data samples based on the acquired physiological signals. It should be noted that the final results presented were the maximum obtained after five repetitions of running the clustering exercise.

**TABLE 8:** Results of EHG unsupervised learning exercise

Model	Preterm (%)	Term (%)	Overall accuracy (%)
GMM	n/a*	n/a*	n/a*
K‐Means	88	54	71

* n/a represents a model error obtained during the running of the GMM

**TABLE 9:** Results of MHR unsupervised learning exercise

Model	Preterm (%)	Term (%)	Overall accuracy (%)
GMM	87	25	56
K‐Means	74	42	58

**TABLE 10:** Results of FHR unsupervised learning exercise

Model	Preterm (%)	Term (%)	Overall accuracy (%)
GMM	98	37	67
K‐Means	53	50	51

#### 3.2.1 EHG

The results of the EHG unsupervised learning exercise can be seen in Table 8, with an overall clustering result of 71 %. It can be seen that the best cluster assignment was that of the cluster containing the preterm samples, where an accuracy of 88 % was obtained, while the term cluster produced a slightly lower accuracy of 54 %. This shows reasonable results for a fully automated partitioning of the various states of pregnancy, albeit with a degree of bias towards the labelling and partitioning of data in the preterm cluster.

That being said, working with the law of ergodicity and the overall sample partitioning accuracy, a supervised learning algorithm robust to noisy labels and outlier detections could be utilised towards building supervised prediction models based on the labels provided by the K‐Means model as part of a multi‐stage model building stage.

#### 3.2.2 MHR

For the case of the maternal heart rate, the preterm cluster appears to be the best sorted cluster for both clustering algorithms, with a relatively lower accuracy when compared with the uterine contraction signals, therein highlighting the compatibility of the EHG signals with the applied clustering algorithm. For the case of the GMM, the preterm cluster is partitioned with a high accuracy, as can be seen in Table 9, while the term cluster dips heavily and suffers from a poor clustering result, hence the final accuracy.

In the case of the K‐Means method, the same trend is shown with the higher accuracy belonging to the partitioning obtained for the preterm cluster. Although the clustering of the term cluster provided a relatively higher accuracy in comparison with the results from the GMM—hence the slightly improved score—the accuracy figures are considerably lower than that of the supervised learning exercise from the prior section, therein suggesting that the unsupervised learning method may not suffice for the maternal heart rate‐based separation of the preterm and term pregnancies.

#### 3.2.3 FHR

The foetal heart‐rate clustering exercise to predict preterm produced a slightly improved set of results in the case of the GMM, where the trend continued, as seen in Table 10. In particular, a high partitioning accuracy was obtained for the preterm cluster at 98%, alongside a slightly lower figure for the term data class, with an overall accuracy of 67%. For the case of the GMM, this provides signs to suggest that the clustering methods could have some potential towards being utilised for an automated class labelling. In contrast, the K‐Means produced lower accuracy metrics for this clustering exercise while recording a slightly higher metric for the preterm cluster, as was seen to be the trend with the acquired physiological data.

## 4 CONCLUSIONS AND FUTURE WORK

The preterm epidemic is one which has been seen to cause a large number of deaths in infants, as reported by the WHO, and which brings not only health implications with significant financial impacts to the economy. There continues to be active research for effective and optimal means towards the reliable prediction of a premature birth.

The use of uterine contraction signals alongside machine models is a method that has seen a substantial amount of research, yielded positive results, and forms a part of what has been investigated as part of this paper. The use of EHG, FHR and MHR physiological signals were investigated to observe the extent to which they can be used to predict the delivery of a premature foetus in a group of Hispanic patients, who were in active labour. As part of the prediction exercises, the pattern recognition models used involved the utilisation of supervised learning models which are reliant upon the supply of labels; and also unsupervised learning which represents an advanced form of intelligence that is capable of self‐learning from data.

In terms of the results for the array of supervised learning models tested it was seen that the LSDL provided a considerable improvement in the prediction accuracy. In the case of the EHG, the LDA and LR provided the best prediction metrics. For the MHR, the LR and QSVM produced the best prediction metrics, while in the case of the FHR, the LDA and LR produced the best metrics. Thus, either one of these physiological signals can be utilised towards the prediction of preterm deliveries with women who are in active labour. However, the heart rate signals were seen to use a much smaller time window and therein less data, and their models can also be expanded towards the monitoring of cardiac‐based diseases from the same signal source. For the unsupervised learning, the K‐Means results showed that the EHG data could be partitioned and sorted as part of a self‐learning setup, whereas for the case of the MHR and FHR a relatively low clustering result was achieved, therein showing its ineffectiveness in self partitioning of information from these signals.

Further work in this area would involve continuous research in the use of unsupervised methods towards feature extraction, along with the further tuning of the parameters of the unsupervised learning models used in this work for potential improved model performance. Along with an application of a wider range of unsupervised learning techniques where possible, that is, Spectral Clustering, and further validation exercises of the designed supervised learning models. [43]. In addition to this, the use of patient health records acquired through pregnancy all the way towards delivery would be utilised as a means towards the investigation of non‐physiological signal methods towards the prediction of preterm births during labour [14, 55]. The main limitation associated with this study is the constrained amount of data which pertains to a single ethnicity. Which although provides pilot information to better interpret physiological manifestations, cannot be generalised due to the sample size used.

## CONFLICT OF INTEREST STATEMENT

The authors declare no conflict of interest.

## PERMISSION TO REPRODUCE MATERIALS FROM OTHER SOURCES

None.

## Data Availability

Data is available upon reasonable request from the authors.
